# High-Fat Diet and Female Fertility across Lifespan: A Comparative Lesson from Mammal Models

**DOI:** 10.3390/nu14204341

**Published:** 2022-10-17

**Authors:** Chiara Di Berardino, Alessia Peserico, Giulia Capacchietti, Alex Zappacosta, Nicola Bernabò, Valentina Russo, Annunziata Mauro, Mohammad El Khatib, Francesca Gonnella, Fani Konstantinidou, Liborio Stuppia, Valentina Gatta, Barbara Barboni

**Affiliations:** 1 Faculty of Bioscience and Technology for Food, Agriculture and Environment, University of Teramo, 64100 Teramo, Italy; 2 Institute of Biochemistry and Cell Biology (IBBC), National Research Council, A. Buzzati-Traverso Campus, via E. Ramarini 32, Monterotondo Scalo, 00015 Rome, Italy; 3 Department of Psychological Health and Territorial Sciences, School of Medicine and Health Sciences, “G. d’Annunzio” University of Chieti-Pescara, 66100 Chieti, Italy; 4 Unit of Molecular Genetics, Center for Advanced Studies and Technology (CAST), “G. d’Annunzio” University of Chieti-Pescara, 66100 Chieti, Italy

**Keywords:** high-fat diet, obesity, female fertility, hypothalamic–pituitary–gonadal axis, ovarian cycle, puberty, oocyte meiotic competence, fertilization, embryo development, pregnancy rate

## Abstract

Female reproduction focuses mainly on achieving fully grown follicles and competent oocytes to be successfully fertilized, as well as on nourishing the developing offspring once pregnancy occurs. Current evidence demonstrates that obesity and/or high-fat diet regimes can perturbate these processes, leading to female infertility and transgenerational disorders. Since the mechanisms and reproductive processes involved are not yet fully clarified, the present review is designed as a systematic and comparative survey of the available literature. The available data demonstrate the adverse influences of obesity on diverse reproductive processes, such as folliculogenesis, oogenesis, and embryo development/implant. The negative reproductive impact may be attributed to a direct action on reproductive somatic and germinal compartments and/or to an indirect influence mediated by the endocrine, metabolic, and immune axis control systems. Overall, the present review highlights the fragmentation of the current information limiting the comprehension of the reproductive impact of a high-fat diet. Based on the incidence and prevalence of obesity in the Western countries, this topic becomes a research challenge to increase self-awareness of dietary reproductive risk to propose solid and rigorous preventive dietary regimes, as well as to develop targeted pharmacological interventions.

## 1. Introduction

Core tasks of the female reproductive system are the specialization of a totipotent oocyte and the tunable release of ovarian hormones to trigger pregnancy. Physiologically, both these functions are cyclically activated under the hypothalamus–pituitary–ovary axis control leading to the release of endocrine and paracrine factors that synergically control the somatic and germinal compartments of the ovarian follicle [1]. However, several factors can dysregulate the reproductive function at different levels by determining female infertility. Some of these factors are closely connected to endocrine dysfunctions or to pathologies affecting the reproductive organs, whereas others are determined by multifactorial influences, most of them related to lifestyle. In this context, diet, including hypercaloric nutrition, may result in metabolic derangements and in body weight increase which promote obesity, an increase in ovarian dysfunction, and the risk of infertility. Indeed, obesity perturbates the hypothalamic–pituitary–ovarian axis dialogue through an inhibitory action exerted by the adipose tissue that has a recognized critical role in maintaining the endocrine/paracrine control of the gonads, with the aim of synchronously regulating the follicle–oocyte development over time until ovulation [2,3]. To date, many research studies that have clarified the mechanisms behind high-fat-related infertility in mammals were carried out in experimental animal models [4,5]. However, to date, multiple methodological differences, such as the length of high-fat diet exposure and the percentage and the typologies of the used fats, have severely limited the identification of the mechanisms interfering with female fertility in mammal models [6]. Thus, a complete depiction of the role of nutrition on fertility is still needed. The most salient gaps in the current knowledge include the minimization of the methodological differences in high-fat diet protocols that have to be validated and the testing of the most consistent findings in randomized clinical trials [7].

Based on this premise, the purpose of this systematic review is to discuss the effects of a high caloric food intake on female reproductive function using a comparative approach amongst mammals during the overall reproductive life cycle. Specifically, it focuses on the main morphological/functional elements affecting three key and interconnected levels: (1) hypothalamus–pituitary–endocrine control, (2) ovarian follicle growth and selection, and (3) oocyte developmental competence acquisition and expression.

## 2. Materials and Methods

### Bibliographic Search Methods

The present systematic review was carried out following the Preferred Reporting Items for Systematic Review and meta-analysis (PRISMA) Statement 2020 Checklist Guidelines (accessed on 1 February 2022 http://www.prisma-statement.org/).

Scientific literature published in the peer-reviewed international index Advanced Search of Web of Science [v.5.35] “Core collection” archive (accessed on 1 February 2022 https://apps.webofknowledge.com/WOS_AdvancedSearch) was considered, using the following key words: “high fat diet*”, “female”, “endocrine”, “puberty”, “reproduction”, “folliculogenesis”, “fertility”, “ovulation”, “oocyte”, and “embryo development”.

“TS” was used as a Field tag, “AND”, ”OR” and “NOT” were used as Boolean operators.

The keywords were combined to elaborate each of the three main paragraphs, as follow:

(1) List: “High fat diet influence on female reproductive endocrine control”

((TS = (high fat diet*)) AND TS = (female)) AND TS = (endocrine)

((TS = (high fat diet*)) AND TS = (female)) AND TS = (puberty)

((TS = (high fat diet*)) AND TS = (female)) AND TS = (reproduction)

Matched list: 590 publications;

(2) List: “High fat diet affecting ovarian folliculogenesis”

(TS = (high fat diet*)) AND TS = (folliculogenesis)

((TS = (high fat diet*)) AND TS = (fertility)) NOT TS = (male)

((TS = (high fat diet*)) AND TS = (ovulation)) NOT TS = (male)

Matched list: 515 publications;

(3) List: “High fat diet impact on oocyte developmental competence”

(TS = (high fat diet*)) AND TS = (oocyte)

(TS = (high fat diet*)) AND TS = (embryo development)

Matched list: 391 publications.

Only publications written in English were considered. The analyzed articles, which examined the correlation between high-fat diet and/or obesity and female fertility, in terms of folliculogenesis, steroidogenesis, and oogenesis, were published between 1990 and 2022. The eligibility of the studies primarily relied on titles and the corresponding abstracts. Except for the articles regarding obesity, the research articles that did not specifically concern a “fat-rich diet” and “reproduction” were excluded. The non-mammalian studies or studies using male animal models were finally discarded.

Considering the above, each list was then matched, and the initial number of titles in accordance with the search keywords were estimated to be 1329. Duplicates were removed. The complete manuscripts were then retrieved for all the selected documents, and the final inclusion was made after a thorough examination.

**Figure 1:**
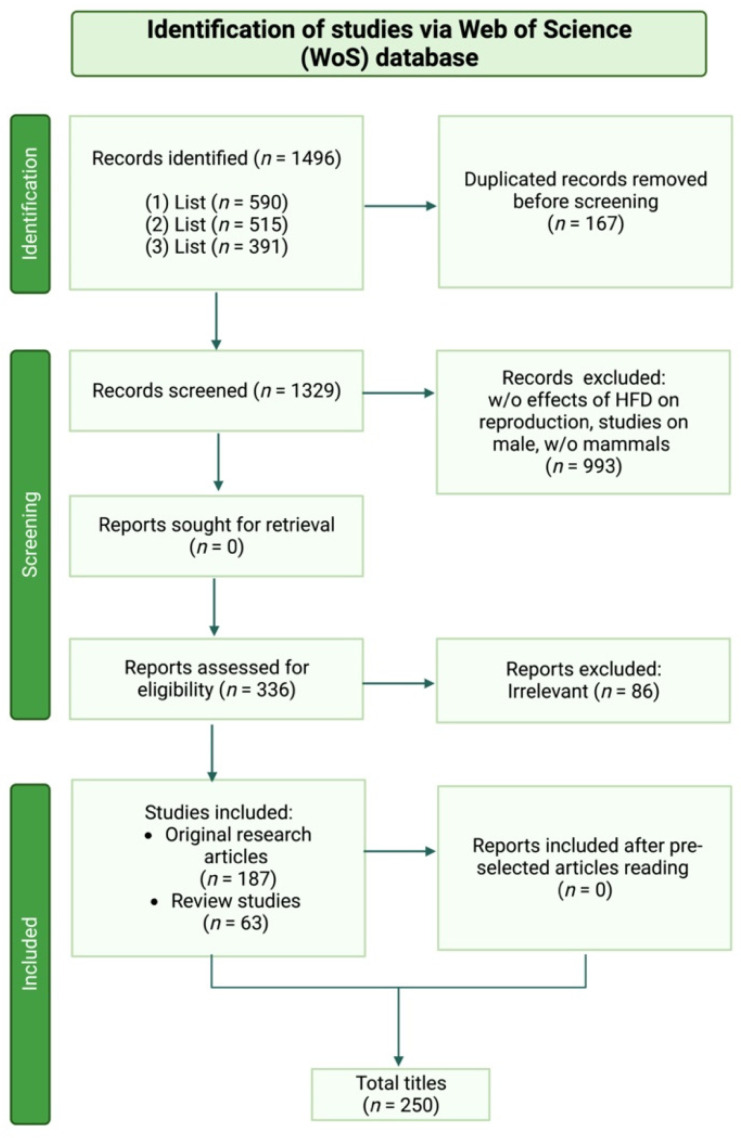
PRISMA flow diagram. The diagram shows the systematic process adopted to include papers captured by the literature search. Preferred Reporting Items for Systematic Review and meta-analysis” (PRISMA) Statement 2020 Checklist Guidelines were followed. Image created with Biorender.com.

Only original research articles (*n* = 187) were selected to document the effect of a high-fat diet on specific mammalian models and to assess their reproductive performances. The review studies (*n* = 63) were only included with the aim to better support and discuss the acquired data. Finally, 250 publications met the inclusion and exclusion criteria (Figure 1).

## 3. High-Fat Diet Influence on Female Reproductive Endocrine Control

Focusing on the complex mechanisms controlling female reproduction, it is widely known that the ovarian cycle is orchestrated by the hypothalamus–pituitary–ovary axis dialogue [8,9,10]. Of note, the success of the female reproductive system is characterized by an interaction between the neuroendocrine and the endocrine signaling produced along the gonadotropic axis [8,11,12]. This cross talk is tightly regulated by evolutionary conserved mechanisms sensing and combining environmental and nutritional cues [13].

The female gonads play a pivotal role in driving the synergistic and functional specialization of folliculogenesis and oogenesis processes by mediating the release of sex hormones. These act on the systemic feedback control on the hypothalamus–hypophysis axis, influencing the development of the female secondary sexual characters and sustaining the gestation [3]. The main biological aim of the gonadal activities in mammals is to lead the specialization of a competent cell, the oocyte, that once fertilized can support both the embryo and fetal development and generate a new vital organism. To lead this process, known also as oogenesis, the female gonads enroll cells surrounding the oocyte, which constitute the somatic compartment of the ovarian follicle unit. Specifically, a finely regulated release of endocrine and paracrine factors, acting on and released from the theca and granulosa cell layers of the follicle, are required for the completion of a synchronous follicle–oocyte development during the estrous or menstrual cycle in mammals [14,15,16].

Obesity caused by a high-fat diet has been associated with estrous or menstrual failure depending on dysregulated endocrine mechanisms [3]. These lead to a defective release of crucial reproductive hormones resulting in an irregular reproductive cycle [6].

Nevertheless, in apparently normal fertility conditions, the hormone release mechanism could also be impaired. As an example, in patients with regular reproductive cycle, during the follicular phase, lower circulating levels of gonadotropins, estradiol, and inhibin were observed. This suggests an intrinsically inhibitory effect of the overweight condition on the release of these hormones through adipose tissue-mediated signaling [3]. The adipose tissue acts as an endocrine organ that releases several bioactive molecules, known as adipokines (i.e., leptin, adiponectin, resistin, visfatin, retinol-binding protein 4, and vaspin). The production of most adipokines is upregulated in obesity [17]. This huge amount of pro-inflammatory proteins causes a chronic low grade of metabolic inflammation which affects the integrity of the hypothalamic–pituitary–gonadal (HPG) axis both centrally, at the hypothalamic–pituitary level, and peripherally, acting on the gonads [3].

In detail, at a systemic level, high adipokine production causes an impaired GnRH pulse due to an overstimulation of kisspeptin neurons. At the pituitary level, there is an anomalous release of gonadotropins which is reflected locally in increased androgen production which ovarian cells fail to fully convert into estrogen [3].

In view of these facts, the following sections aim to collect evidence on the role of a high-fat diet in the alteration of gonadotropins and steroid hormones’ release and how their altered secretion can either directly or indirectly give rise to infertility disorders during the different reproductive and developmental stages: transition into puberty and cyclical ovarian activity in adulthood.

### 3.1. Transition toward Puberty

Recent studies have shown that obesity and premature puberty are closely connected. This correlation seems to be confirmed by the evidence collected to date on obese children and on different experimentally induced obesity animal models [18,19,20]. During puberty, mammals develop secondary sex characteristics, accelerate a linear growth, and gain reproductive functions. All these events are regulated mainly by the central nervous system through leptin signaling [21]. Indeed, evidence collected from the past decades suggest that high circulatory levels of leptin facilitate the onset of puberty by impacting the central kisspeptin/G protein-coupled receptor (GPR54) system [22,23]. Comparative investigations on physiological changes due to the exposure to a high-fat diet using female rats demonstrated that this kind of diet can bring puberty forward by accelerating the luteinizing hormone (LH) pulse frequency and by upregulating the central expression of kisspeptin. These findings suggest that obesity may have a long-term impact on reproductive functions by impairing the estrus cyclicity from the pre-pubertal stage to puberty [19,24].

Importantly, as demonstrated by studies in rodent models, high-fat diet exposure during prepubescence has been reported to increase the probability of developing metabolic disorders which are closely linked to the development of PCOS later in life [25,26]. Nevertheless, several research studies conducted on rats have shown that an early exposure to a high-fat diet has more drastic consequences on the metabolism compared to exposure during adulthood [27].

Several aspects remain to be investigated to identify other events that accelerate puberty in response to high-fat consumption with diet. These key aspects might help to define predictive puberty markers to be clinically tested for prevention treatment purposes [22].

### 3.2. Reproduction in Adulthood

High-fat-diet-induced obesity might influence reproductive performance when puberty occurs at different levels during the reproductive cycle or pregnancy [6] by affecting events related to folliculogenesis, oogenesis, endometrial formation, embryogenesis, and fetal development [22,23].

As mentioned above, the metabolic imbalance triggered by a high fat intake increases the likelihood of encountering metabolic-related diseases in adulthood. Specifically, hyperglycemia, hypertension, insulin resistance, dyslipidemia, and endocrine dysfunctions are the main factors triggering metabolic disorders leading ultimately to fertility problems. To date, different animal models of high-fat diets have been used to assess the effect of induced metabolic defects on fertility failure.

Obesity induced by high-fat consumption in female Ossabaw mini-pigs has been associated with altered metabolic parameters such as hyperglycemia, hyperandorgemia, hypertension, insulin resistance and dyslipidemia, and impaired folliculogenesis due to a dysregulated production and release of LH, FSH, and progesterone [28,29]. Hyperandrogemia has been also documented in overweight women displaying an exacerbation morbidity associated with PCOS [3,30]. Using a rat model, Bazzano and colleagues demonstrated a robust model of high-fat diet for experimental animals based on consumption of tasty but unhealthy food products (e.g., hot dog and muffins) [31] enabled fatness and hyperglycemia by maintaining unaltered serum levels of triglycerides, cholesterol, or C-reactive proteins. In these animals, an alteration of the ovarian function led to prolonged diestrus phases, decreased serum estradiol concentrations, increased presence of follicular cysts, increased number of antral atretic follicles, and decreased number of pre-antral and pre-ovulatory follicles and was linked to a defective ovulatory process [32].

Additional studies conducted in mice proved the negative impact of obesity on the ovarian function [33,34] with different effects on estrous cyclicity. Bermejo-Alvarez and colleagues demonstrated how high-fat diets impaired mice fertility by altering the estrous cyclicity, exhibiteded as either blocked or prolonged estrous cycles depending on body weight gain [33]. Zhou et al., instead, reported shortened estrous cycles in high-fat fed conditions [34]. Several research studies tried to identify the molecular events involved in cyclicity dysregulation, finding the transcriptional program that is necessary to drive follicular and steroidogenic processes [26] and the accumulation of energy sources for sustenance of a proper oocyte maturation both compromised [35,36].

The origin of the estrous cycle impairment due to obesity may also recognize a pituitary dysfunction. This evidence was collected on non-human primates, where mildly elevated levels of androgens, coupled with a high-fat diet, were proved to lead to functional disturbances in the neuroendocrine, ovarian, and metabolic systems, inducing a PCOS-like phenotype in females. Indeed, these animals showed an insulin over-secretion that promoted androgen synthesis and release in theca cells by directly improving the effectiveness of LH and indirectly increasing its pulse amplitude. This significantly higher LH pulse frequency was associated with altered follicular dynamics during the late developmental follicular phases in which delay or suppression of dominant antral follicles’ selection was reported in combination with an increased recruitment of small antral follicles [23].

Dysregulation during the estrous and menstrual ovarian cycle also results in gestation defects in adult females, specifically leading to adverse effects on: (1) fetal annex development, (2) initial size of primordial follicles pool in the fetal ovaries, and (3) defects in ovarian follicle growth and development of the offspring.

Maternal obesity can have adverse effects on the differentiation, development, and function of the major fetal annexes along with long-term deleterious reproductive consequences on the ovarian follicular growth and development in the offspring [18,37,38]. Evidence collected from different mammal models demonstrated that placental insufficiency induced by the mother’s exposure to a fatty-acids-rich diet during pregnancy impaired fetal development in rodents [38,39,40,41,42,43,44] and in pigs [29,45].

Another phenomenon constituting a negative impacting element on female reproductive systems consisted of a reduction in the number of primordial follicles in the fetuses and neonates ovaries as reported in both rodents [38,41,42,46] and rabbits [18]. However, the effects of maternal nourishment on the initial size of the primordial follicles pool and the mechanism of actions involved are not conclusive, but increasing evidence in mammalian models seems to correlate the diet to ovarian reserve establishment during the fetal lifetime [16,42,47].

Defects in ovarian follicle development in the offspring have also been linked to a maternal high-fat diet. Importantly, exposure to some environment-derived factors commonly used to ameliorate obesity by means of their estrogen-like activity can worsen reproductive performance of the offspring, impairing fetal folliculogenesis. As an example, a recent study conducted in obese female mice reported that fetus exposure to Genistein, an isoflavone used for obesity treatment in humans and animals, exerted adverse effects on the female offspring with obesity, induced by a high-fat diet, significantly prolonging the estrus cycle, disrupting sex hormonal balance and ovarian follicle development [44]. In a similar way, endocrine disruptors such as di-(2-ethylhexyl) phthalate (DEHP), associated with an obesity condition derived from maternal exposure to a high-fat diet, synergistically disrupted mouse fetal oogenesis, leading to synapsis defects in the meiosis process and affecting folliculogenesis of the offspring [48].

The decrease in the number of primary, secondary, and tertiary follicles have also been observed as a result of a defective folliculogenesis in the offspring ovaries, together with morphometric follicle alterations including smaller ovaries [3,39,41,42,45].

Mammals’ offspring are also influenced by the mother’s diet during the lactation period [49]. The mechanisms by which maternal diet and the metabolic profile shape the perinatal environment remain largely unknown. However, recent evidence suggests that maternal obesity during gestation and lactation may increase the risk of exposed individuals developing a metabolic syndrome later in life, impairing puberty in female offspring [50]. Moreover, parentally acquired metabolic changes can alter the metabolic health of the filial and grand-filial generations [41,43,51]. Notably, these effects are strictly dependent on obesity-related accumulation of reactive oxygen species (ROS) and stress increase which cannot be properly managed, thus promoting persistent cell damage and a chronic low-grade inflammatory state which the offspring will carry later in life [52].

**Figure 2:**
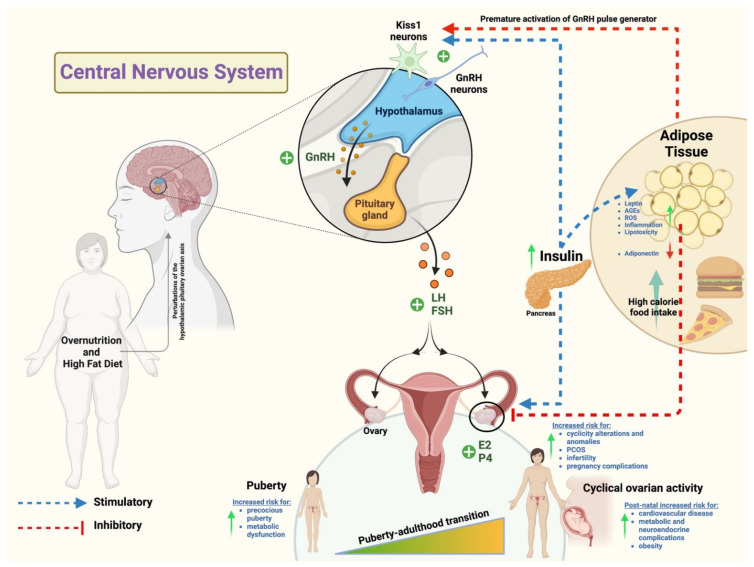
Impact of a high-fat diet on the female reproductive neuro–endocrine mechanism during puberty–adulthood transition. High-fat caloric intake leads to adipose tissue accumulation resulting to an increased systemic release of adipokines. High adipokine production causes an impaired GnRH pulse due to an overstimulation of kisspeptin neurons, which in turn affects the release of the gonadotropins FSH and LH from the pituitary gland and the secretion of E2 and P4 from the ovary. In addition, high insulin levels, characterizing a diet with high fat intake, increase GnRH pulse amplitude in the hypothalamus leading to increased LH secretion from the pituitary. These alterations negatively impact reproductive functions anticipating puberty onset in prepuberal subjects and affecting cyclical ovarian activity during adulthood. List of abbreviations included in the figure: gonadotropin releasing hormone (GnRH); follicle stimulating hormone (FSH); luteinizing hormone (LH); estradiol (E2); progesterone (P4); polycystic ovary syndrome (PCOS); reactive oxygen species (ROS); advanced glycation end products (AGEs). Image created with Biorender.com.

An overall summary of the information described above is reported in Figure 2.

## 4. High-Fat Diet Affecting Ovarian Folliculogenesis

The complex mechanisms regulating ovarian folliculogenesis reflect their susceptibility to maternal physiological suitability. Overweight mothers fed high-fat diets are an outstanding model of a biological condition in which the ovarian follicular environment may be compromised [53]. More in detail, uncontrolled fat intake may affect both germinal and somatic follicle compartments, with the incidence of obesity earlier in life constituting a further risk for subsequent oocyte development [3]. The gradual increase of adipose tissue determining severe hyperandrogenism and hyperleptinemia has been associated with altered follicular fluid composition and compromised granulosa cell signaling affecting ovarian performance in early and late stages of follicle development [6,54,55,56,57]. Below, the effects of high-fat consumption, collected form the systematic literature survey, are classified and listed according to each ovarian follicle developmental stage.

### 4.1. Effect on Early Ovarian Folliculogenesis

Early ovarian folliculogenesis is characterized by two sequential events allowing proper follicle development. Firstly, the pool of primordial follicles is expected to endure one of three possible fates: (1) remain quiescent, (2) die directly from their dormant state, or (3) be activated [15]. Secondly, if activated, follicles are assumed to proceed to the preantral stage [16,58].

Notably, deregulated pathways affecting the general equilibrium among dormancy, activation, and death of primordial follicles are the mammalian target of rapamycin (mTOR), phosphatidylinositol 3-kinase/Protein kinase B/Forkhead Box O3 (PI3K/AKT/FOXO3) and Sirtuin1 (SIRT1) [15,59]. These paths are responsible for the regulation of key cellular processes, such as proliferation, differentiation, and survival, and are the result of bi-directional cross talk between somatic and germinal compartments [15,59].

Briefly, the recruitment of primordial follicles is due to the activity of mTOR in pre-granulosa cells (pGCs) and the PI3K/AKT path in the oocyte. Activation of mTORC1 signaling in pGCs leads to their differentiation into granulosa cells (GCs) which become able to stimulate dormant oocytes through KIT ligand (KITL) secretion. Binding of KITL to KIT on the surface of the oocytes activates a PI3K/AKT/FOXO3 signaling cascade in dormant oocytes and the subsequent stimulation of the dormant oocytes resulting in primordial follicle activation [60,61]; mTOR and PI3K signaling are also regulated by the activity of SIRT1 in both pGCs, where it links to the differentiation of pGCs into GCs and oocyte leading to its activation. At a molecular level, it mediates deacetylation-dependent activation of FoxO3 [62,63], and it acts as a cofactor regulating transcriptional activation of mTOR and PI3K genes [64].

Any perturbation of these cascades might drive dysfunctional primordial follicle activation, impacting the primordial follicle reserve and ultimately leading to premature ovarian failure (POF).

Evidence on the role of the molecules that constitute these paths is obtained from transgenic models. Transgenic mouse models with constitutively active FoxO3 demonstrate that the overexpression of FoxO3a impaired oocyte growth and folliculogenesis, leading to female infertility [65]. Accordingly, John et al. [66] reported that PI3K/AKT dependent overactivation of FOXO3 induces over-activation of primordial follicles [67,68], and AKT knock-out mice showed decreased primordial follicle reserve [69]. SIRT1 knock-in mice exhibited a protective role for Sirt1 in preserving ovarian reserve [62].

Similar effects were observed in animal studies using high-fat dietary regimens. Specifically, high fat intake was identified as a negatively impacting element, whereas a dietary restriction regimen was associated with the reversal of ovarian dysfunctions [70].

Molecularly, the abovementioned paths work as energy sensors triggering the response to any metabolic environmental shift. As some examples, uncontrolled activation of mTOR path and concomitant suppression of SIRT1 signaling due to an excess of high fat intake in rat models was found to be associated with an acceleration of ovarian follicle development and follicle loss [71,72] leading to POF. Furthermore, high-fat-diet-induced obesity mouse models showed impaired early follicle growth and viability through dysregulation of PI3K, AKT, and FoxO3 gene expression pattern [41,73]. In addition, retardation of early follicular development was observed in mice on a high-fat diet regimen because of decreased AKT-dependent phosphorylation of FOXO3 resulting in its over-activation [74]. Accordingly, another study showed that downstream FOXO3 target genes, such as cyclin p21 and cyclin p27, are upregulated in high-fat-diet mice conditions, inducing cell cycle arrest, finally leading to GCs apoptosis during the early phases of follicular growth [56].

Deregulation of the proliferation and survival pathways mentioned above also leads to an alteration in the morphological and functional structure of the GCs, affecting the follicular transition from the primordial stage. Firstly, acquired data show that altered follicular microenvironment in overweight or obese females might exert a detrimental effect on GCs morphology by leading to uneven cells layer (in mice: [75,76,77]; in ruminants: [78]; in rabbits: [18]; in humans: [5,79]) and to the accumulation of intracellular lipids that bind to proteins forming lipid droplets, thus causing lipotoxicity and consequent cumulus cell apoptosis (in mice: [47,56,80] and in humans: [79]). Of note, the lipotoxic effect is mediated at a systemic level by high levels of circulating leptin in high-fat-diet conditions [54]. Specifically, increasing the quantity of leptin derived from the accumulation of adipose tissue determines a systemic endocrine imbalance which has direct effects on the reduction of primordial follicular reserve in rat [71] and mouse [73,81,82,83] models. Secondly, the intake of a high-fat content with diet results in a negative impact on GCs paracrine signaling, determining an alteration of the follicular fluid (FF) composition with increased levels of triglycerides, free fatty acids, and glucose. This ultimately results in the inhibition of estradiol (E2) production which is responsible for an impaired primordial follicle assembly and development from primordial to preantral follicle stage as demonstrated in several animal models, such as mice [36,56,70], rats [25,26,32,71,84], pigs [28,29,85], and primates [30,57,86,87].

Overall, the dysfunction of any event encompassing early follicular development determines an impaired progression into more advanced stages. The signaling involved in the impairment of early folliculogenesis deserves to be further explored, especially because defects due to a diet rich in fat can recur in future generations leading to the inheritance of the same defects in the reserve of the ovarian follicle.

### 4.2. Effects on Late Follicle Development under Physiological and Hormonal-Induced Conditions

While early folliculogenesis appears to be controlled by the ovarian microenvironment and intrafollicular paracrine and autocrine signaling, late mammal ovarian folliculogenesis is gonadotropin-dependent and is controlled by multiple factors and physiological events that drive the proliferation of GCs, the development of the thecal layer, and antrum formation, three integral processes that allow preantral follicles to reach the antral stage [88]. In the late preantral follicle stage, the theca cell layer forms and can be stimulated by LH to synthesize androgens, which diffuse into the GCs as an estrogen precursor useful for the completion of a synchronous follicle–oocyte development. The theca layer participates to develop the blood vasculature, providing nutrients as the follicle expands and the follicular antrum forms.

A high-fat diet negatively impacts these processes, predominantly through an alteration of the insulin signaling pathway [16,28,45,46,83,89] due to an excessive accumulation of adipokines that characterize females fed with a high-fat diet [3]. Importantly, it has been shown that insulin has gonadotrophic actions in the ovary [90,91]. Physiologically, insulin participates in ovarian steroidogenesis through its own receptors found in granulosa and thecal cells [90,92,93]. Indeed, it stimulates the theca cells to produce androgens and exert a growth stimulatory effect on GCs cells, thus priming the production of estradiol [3]. Overstimulation of the insulin pathway due to a high-fat diet reinforces the activity of LH on theca cells causing excessive androgen production which in turn contributes to the dysregulation of GCs differentiation in female mice [36,41,46,70,74,75,94,95,96,97], rats [19,25,26,32,42,71,72,84,98], ruminants [20], pigs [28,29,45], rabbits [18], humans [79], and non-human primates [57,86,87], compromising the follicular progression [23,99,100]. As a consequence of GCs dysregulation, a poor follicular vascularization, which at a molecular level is attributable to dysfunctional behavior of the angiogenic factors, such as the vascular–endothelial growth factor A (VEGFA) [101,102,103], vascular–endothelial growth factor receptor 1 (VEGFR1) [104], PDGF [105], transforming growth factor beta (TGF-β) [106,107], basic fibroblast growth factor (bFGF) [108], angiopoietin 1 and 2 [105,109], and placenta growth factor (PGF) [110,111] in humans, has also been reported.

Yang et al. showed that a key factor in the disruption of VEGF-induced follicular angiogenesis occurring in a diet rich in fat is the adipokine Interleukin-10 (IL-10). The authors demonstrated that female mice fed high-fat feed showed an increased production of IL-10 in visceral fats, contributing to a reduction of antral follicles number and size [87,94].

Thus, the premature luteinization, the suppression of ovarian angiogenesis, and the consequent follicular arrest at later stages of antrum formation ultimately results in estrus and menstrual cycle disorders and anovulation (bovine: [112]; non-human primates: [87,113]; mice: [35,48,70,74,94,97,100,114,115,116,117]; sheep: [78,118]; rats: [32,98]).

Overall, features such as hyperandrogenemia, hyperinsulinemia or insulin resistance, and the number and morphology of antral follicles constitute the main determinants for the dysregulation of late folliculogenesis events and might be considered as predictive markers of ovarian functionality along with the AMH test [119,120] for the implementation of the current clinical protocols for fertility status diagnosis. The full comprehension of the abovementioned events will also allow us to draw suitable therapies to face ovarian failure [54], especially in those females who need a superovulation treatment to increase their reproductive performance.

Systematically, high-fat-diet-induced overweight females have lower outcomes following fertility treatments (also known as assisted reproductive technologies/ART) than the healthy-weight population. They responded poorly to ovulation initiation and require increased doses of gonadotropins and prolonged rounds of treatments for follicular development and ovulatory cycles. These effects have been shown in the literature with different mammal models (pigs: [29]; mice: [121,122]; non-human primates: [57,86,87]; human primates: [5,79]).

Indeed, ovarian stimulation for ART yields fewer preovulatory follicles leading to the collection of a reduced number of oocytes (mice: [96,114]). Conversely, other studies demonstrated that although the collected number of oocytes derived from preovulatory follicles and the percentage of meiotic competent oocytes after hormonal stimulation between control and high-fat-diet-fed mice were comparable, the key difference was correlated to the fertilization rate. Therefore, mice with high-fat caloric intake presented a lower fertilization index in contrast to the control groups [71,97,114], and the embryo quality was weakened, causing a low pregnancy rate and greater risk of early pregnancy loss [3].

### 4.3. Effects on Ovulation Rate

The LH surge triggers a connected network of events within the follicular microenvironment that plays a pivotal role in driving ovulation and the related processes such as: (1) ovulatory transcriptional program induction, (2) the activation of processes involved in ovulation dependent on immune-cell recruitment, and (3) lipid metabolism and oocyte developmental competence supply. Several research studies showed that the diet regimen impacts the hormonal signaling implicated in ovulation, strongly affecting reproductive performance [6,15,59,123]. Firstly, regarding gene activation, it has been shown that a high-fat diet causes a decreased ovarian expression of those genes implicated in normal ovulation function that are physiologically enhanced in preovulatory follicles, such as Edn2 [36], Errfi1 [36], Tnfaip6 [36], Adamts1 [36], and Cyclooxygenase-2 (COX-2) [32,124]. Their decreased expression corresponds to defective ovulation and reproductive failure, as observed in genetic ablation studies in mice. Edn2 is involved in tightness of the pre-ovulatory follicle causing rupture and release of the oocyte, and when Edn2 is lacking, as shown in knock-out mice, it signifies impaired ovulation [36]. Edn2 is also involved in luteinization, the process needed to form the corpus luteum from the follicular cells. However, studies in rats showed the link between obesity and the lack of a preovulatory surge of progesterone and LH which consequently causes a decrease in the expression of the Edn2 gene [36]. Errfi1 may be important for theca and granulosa cell differentiation and the cumulus–oophorus complex (COC) expansion [125]. Tnfaip6 is involved in forming the extracellular matrix (ECM) during cumulus mucification. It has been shown that female mice lacking Tnfaip6 have no COC expansion, and the oocytes are unable to undergo to fertilization. Adamts1 is also involved in the remodeling of the ECM, as well as the follicular development, and mice lacking this gene display decreased rates of ovulation [126].

COX-2 is a key rate-limiting enzyme in the biosynthesis of prostaglandins which are other key molecules involved in ovulation triggering. It has a role in regulating the gonadotropin-dependent increase of prostaglandins in follicles prior to ovulation. Indeed, defective ovulation and consequent female reproductive failure have been observed upon its genetic ablation in mouse models [127].

Secondly, processes involved in ovulation-dependent immune-cell recruitment are also affected by a high-fat diet. Monocyte recruitment from the blood into the ovary is regulated by the molecular factor MCP-1, whose serum levels are finely regulated under physiological conditions; MCP-1 increases in the FF and in the ovarian stromal cells to assist the ovulatory process [128]. Systemic serum MCP-1 levels are elevated in obesity induced by a high-fat-diet lifestyle where it plays a role in attracting abnormally large amounts of monocytes into the adipose tissue, thus contributing to chronic low-grade inflammation [76,129]. Similarly, high levels of pro-inflammatory (CRP, IL6, TNFα) and anti-inflammatory (adiponectin, IL10) mediators found in FF of obese women due to high-fat diets have been shown to impair the ovulatory mechanism and thus the oocyte developmental competence [130].

Although the process encompassing ovulation-dependent immune cell recruitment dysregulation remains to be fully addressed, it can be supposed that the surplus of monocyte accumulation into the ovaries adversely impacts oocyte quality, GC, and theca cell function, interfering with the normal ovarian ovulatory role. Asemota et al. collected a diminished number of oocytes following superovulation in mice fed with a high-fat diet [76].

Lastly, a correct lipid metabolism is essential to ensure the proper energy level to supply the oocyte maturation and ovulation. Triglycerides are metabolized by lipases that have been localized in cumulus cells as well as oocytes. Fatty acids generated by lipolysis are further metabolized by β-oxidation in mitochondria to produce ATP useful to sustain oocyte maturation. In addition, β-oxidation is influenced by nutritional status, including a high level of ingested fat [131]. Notably, its inhibition in cumulus-enclosed oocytes has been found to prevent the oocytes from eliciting the appropriate hormone-induced meiotic activation and maturation in vitro, causing fertility impairment in the mouse [132,133] model.

A greater understanding of the in vivo regulation of the abovementioned mechanisms could allow us to develop strategies able to improve oocyte developmental potential in domestic animals and alleviate sub-fertility in women.

**Figure 3:**
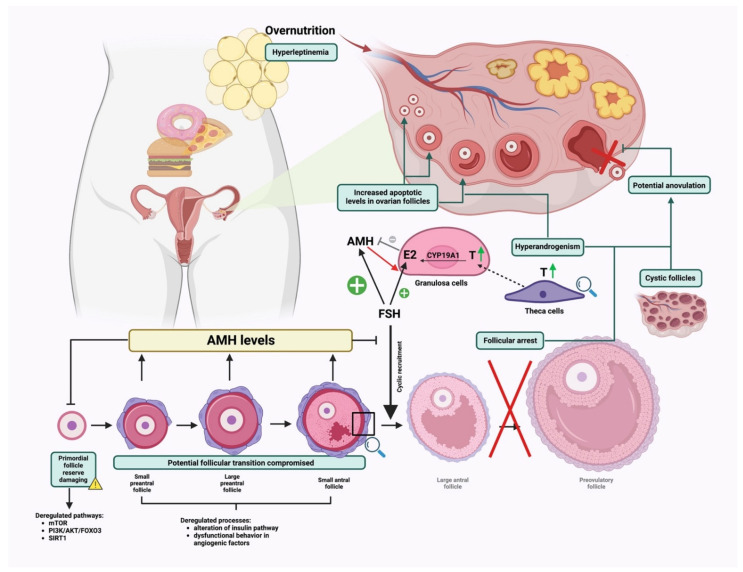
Impact of a high-fat diet on the ovarian folliculogenesis. Hyperleptinemia determines a systemic endocrine imbalance which has deleterious effects on the follicular pool. As a result, processes encompassing each step of the ovarian folliculogenesis (early, late, and ovulation) are affected. In early folliculogenesis, high-fat diet-dependent deregulation of key proliferative and survival pathways (mTOR, PI3K/AKT/FOXO3 and SIRT1) causes defects in the primordial follicle activation process. Alterations of the insulin and the angiogenic pathways together with an increased production of AMH are then responsible for an impaired progression to the next steps of folliculogenesis. In detail, overstimulation of the insulin pathway causes excessive T production (by theca cells) that GCs are not able to convert into the estrogen E2. Dysfunctional behavior in the angiogenic factors leads to poor follicular vascularization. In addition, high AMH levels make the follicles more resistant to the action of FSH, resulting in the inhibition of the follicular maturation up to ovulation. Finally, menstrual cycle disorders and anovulation are consequences of the above-deregulated processes. List of abbreviations included in the figure: the mammalian target of rapamycin (mTOR) phosphoinositide 3-kinases (PI3K); AKT serine/threonine kinase (AKT); forkhead box O3 (FOXO3); sirtuin1 (SIRT1); anti-Müllerian hormone (AMH); follicle stimulating hormone (FSH); testosterone (T); estradiol (E2); aromatase (CYP19A1). Image created with Biorender.com.

The overall flowchart of the presented data is reported in Figure 3.

## 5. High-Fat Diet Impact on Oocyte Developmental Competence

Successful female mammalian reproduction depends on the oocyte quality, which is essential to ensure embryo development after fertilization [134,135,136,137,138]. Oocyte quality is acquired during folliculogenesis as the oocyte grows and during the period of oocyte maturation. Therefore, the processes occurring in the follicular microenvironment play a fundamental role in determining oocyte quality [6,139,140].

During oocyte growth, there are a variety of processes occurring within the cytoplasm of the oocyte that are required to complete developmental competence following fertilization, such as the events that support the level of energy metabolism and signals deriving from the communication between the oocyte and the surrounding follicular cells. Successful completion of these events is collectively referred to as cytoplasmic maturation. An oocyte that has not completed its cytoplasmic maturation is of poor quality and thus unable to successfully complete the maturation step. Consequently, it has an impaired developmental ability.

Perturbations of the abovementioned processes and regulatory factors have a negative impact on the definition of oocyte quality. One of the most impacting trigger factors in Western countries is the habit of consuming a diet rich in fat [6].

### 5.1. High-Fat Diet and Oocyte Quality

During cytoplasmic maturation, both cumulus cells (CCs), which support the oocyte with key products to be used for the generation of ATP [141,142], and FF, which is the collector of these energetic precursors (substrates) [143,144], as well as the molecules of the paracrine signaling between germinal and somatic compartments useful to sustain complete oocyte developmental competence following fertilization, are critical determinants for oocyte quality [142].

In the FF, the principal intracellular substrates useful to produce ATP as a source of energy are fatty acids and pyruvate which are processed in mitochondria by means of oxidative phosphorylation reactions [134].

A high-fat diet was found to determine an excessive availability of these kinds of precursors which in turn causes oocyte quality impairment [134]. The accumulation of intracellular lipids leads to high levels of free fatty acids that are subject to oxidative damage and the formation of cytotoxic and highly reactive lipid peroxides, which ultimately are detrimental to intracellular organelles, particularly the endoplasmic reticulum (ER) and mitochondria [145]. Indeed, healthy oocyte mitochondria require a balance of pyruvate and fatty acid oxidation to maintain a low level of otherwise damaging ROS production [146]. In a recent study authored by Li and colleagues, the expression level of genes involved in mitochondrial function (tim23, tom40, pnpt1, Cox-2, Cyto C, Sox2) was lower in CCs from high-fat-fed mice than that from the control group. The mitochondrial membrane potential decreased, while ROS levels increased [80]. The overall result was an impairment of proper mitochondrial function with an unbalanced use of oxidative phosphorylation cascades.

Recent studies in women [147], goats [148], and mice [55,149] corroborate the detrimental effect of precursor availability in FF resulting from a high-fat diet, showing an accumulation of oxidative stress biomarkers [147,148] and a reduction of antioxidant abilities [147].

Importantly, a high-fat diet might also modify the fatty acids profile as shown in bovine [150] and human [151,152,153] models by inducing an important increase of NEFA levels (such as palmitic acid, oleic acid, and stearic acid) in FF.

Moreover, studies in mice [151,153,154,155,156,157], humans [158], bovines [150,159,160,161,162,163,164,165,166,167,168], pigs [85,169,170,171], and sheep [172] demonstrated that the lipid profile alteration resulted in lipotoxicity in the follicular cells and oocyte. Follicular cell biological functions were affected in their viability, proliferation, ROS production, lipid storage, and ER stress response which ultimately resulted in apoptosis. Compromised oocyte functions corresponded to mitochondrial function, maturation, developmental competence, lipid storage, and ER stress response. The ultimate result was the impairment of the bi-directional communication between the somatic and germinal compartments useful to sustain oocyte fertility potential and to obtain successful ART protocols.

### 5.2. High-Fat Diet Affects Oocyte In Vitro Maturation Performance

Oocyte quality is a crucial factor for successful outcomes in ART procedures. In fact, evidence collected to date shows how oocyte quality defines its potential ability to resume meiosis and undergo meiotic maturation reaching Metaphase II (MII) nuclear stage upon proper hormonal stimulation. Only those oocytes are presumed to be capable of being fertilized [173,174].

Poor oocyte quality caused by high fat intake reflects in maturation defects, as evidenced by several studies involving the application of ART protocols in mammal models.

Indeed, several detrimental effects have been observed during in vitro maturation of oocytes derived from females exposed to high-fat diets, which can be classified into three main categories: (1) yield defects, (2) functional defects, and (3) morphological defects.

Firstly, mouse [46], bovine [20,175], pig [45,85], human [4,79], and non-human primate [57,86,87] models showed that compared to the control group, the high-fat diet group exhibited fewer collected MII oocytes. An increased rate of gametes that were not able to resume meiosis and were thus arrested at Prophase I of the meiotic cell cycle was also observed in mice [33,40,46,48,99,100,114,132,176,177,178] and pigs [45,85], with an incidence of oocyte degeneration [48,99,176,179,180,181] in rats [42] and bovines [182].

Secondly, functional defects including mitochondrial dysfunction, such as increased membrane potential de-regulation inducing oxidative stress, were observed in mice [47,55,80,176,177,178,181,183,184,185] and in bovine [186] models. Notably, the oxidative damage could result in an inability of the oocyte to replicate healthy mitochondria and to replace the damaged organelles, generating an oocyte compensatory response that severely compromises the reproductive energetics of the oocyte during the progression from immature GV to mature MII nuclear stage [177].

Lastly, morphological defects such as disrupted meiotic spindle and chromosome morphology have been reported in several studies in mice [46,48,55,100,117,132,176,177,178,179,183,185,187]. Specifically, these studies showed that exposure to spindle disrupting compounds can impair in oocyte meiotic division and genomic stability, compromising their ability to reach meiotic competence.

Despite advances, further insights into the events responsible for the perturbation of proper oocyte maturation in a high-fat diet are still required to improve current ART protocols for fertility preservation in obese females, as well as to set up therapeutic preventive strategies.

### 5.3. Relation between High-Fat Diet and Fertilization Rate

Following the resumption of meiosis during maturation, mammalian oocytes arrest at MII. Fertilization encompasses three sequential steps, allowing the transition from oocyte to embryo: (1) oocyte–spermatozoon recognition; (2) spermatozoon entry inducing the release of cortical granules, the completion of meiosis, and pronuclear formation; and (3) pronuclei fusion and zygote formation [188,189].

The phenomena that characterize the first two phases are collectively known as “oocyte activation” [190,191,192].

Low rates of fertilization may result from impaired mechanisms involved in the abovementioned steps. Although few studies are available in the literature on the negative impact that a high-fat diet has on these mechanisms, a correlation between this type of regimen and decreased fertalization rate has been widely recognized.

In the first phase, the fertilization performances rely on markers of oocyte quality, as female gametes with cytoplasmic immaturity are unlikely to respond properly to the activation signal provided by the spermatozoa.

Calcium signaling is the universal trigger of oocyte activation in all species studied to date [191], and in mammals, this signal adopts a pattern of brief but periodic increases in calcium [193]. In the context of a high-fat diet, the time-regulated calcium storage and the releasing mechanism results are de-regulated, as shown in mouse models [155]. Indeed, high levels of free fatty acids cause changes in the lipid composition of the membrane of the endoplasmic reticulum (ER) and damages its ability to retain calcium, which in turn alters the cytoplasmic homeostasis [194]. Prolonged calcium release from the ER leads to altered mitochondrial membrane potential and the induction of apoptotic pathways [195,196], as mitochondria are the primary effectors of calcium absorption [197].

The response to spermatozoa signals also depends on the action of cumulus cells surrounding the oocyte. The cumulus cells attract [198], trap [199], and select spermatozoa [200] and prevent the premature hardening of the zona pellucida (ZP) [201], all of which are necessary to start the fertilization process [202]. Considering that the signaling useful for the correct regulation of the proliferation processes and the differentiation of cumulus cells is damaged by a diet rich in fatty acids [137], their biological functions are damaged as well. Several studies have shown an association between elevated levels of free fatty acids in women’s FF and serum and COC with poor morphology during in vitro fertilization [203]. Mouse model studies have consistently confirmed the effects of a high-fat diet on cumulus cell morphology [46].

Furthermore, successful completion of oocyte meiosis following spermatozoon entry requires well segregated chromosomes [204,205]. The fine mechanism that governs the chromosome segregation in an oocyte can be compromised by a diet rich in fatty acids, causing alterations in the meiotic spindle assembly that will result in fertilization failure. This effect has been reported in several studies involving mouse models [46,55,117,132,176,177,178,185]. All the mentioned studies corroborate the hypothesis that the low fertilization rate in mice fed with a high-fat diet might be due to the poor oocyte quality, which was reflected by the disrupted subcellular structures such as chromosomes. Moreover, modifications involving the meiotic spindle assembly are also reflected in an increase in oocytes’ oxidative stress, as proven by the elevated ROS levels recorded [206], causing oocyte fragmentation due to nuclear and mitochondrial DNA damages, often resulting in aneuploidy [134,177,178,179,185]. Pronuclei formation represents another downstream event of spermatozoon entry allowing oocyte activation, and this literature survey found only one study showing that a high-fat diet could interfere with this phenomenon.

In detail, Yamamoto et al. observed a reduction in pronuclear formation in oocytes retrieved upon artificial activation protocols in high-fat-diet mice when compared to the control, suggesting that this type of dietary regimen might deteriorate the induction of the oocyte’s activation [207]. Although specific mechanisms determining adverse effects on oocyte activation in high-fat-diet mice need to be better defined, the authors supposed a crucial role for some lipidemic factors such as omega-3 and omega-6 polyunsaturated fatty acids [207]. This systematic literature survey failed to find data supporting the impact of a high-fat diet on the completion of the fertilization process. This could be the direct effect of the lack of information on the mechanisms by which pronuclei move inwards the fertilized oocyte for their unification in mammals. It can only be hypothesized that a compromission of the dynamic network of microtubules, which is responsible for moving male and female pronuclei from the periphery to the center of the oocyte, is involved [208].

### 5.4. Effects of High-Fat Diet Composition on Embryonic Implantation and Pregnancy Rate

The initial stages of pregnancy, including conception, are crucial in determining the outcome. After the oocyte is fertilized, it then develops into the multi-cellular blastocyst, characterized by an inner cell mass, which will form the embryo, and an outer layer of cells called trophoblasts, which will develop into the placenta [209]. The blastocyst moves freely within the uterine cavity and begins the process of implantation in the uterus. Notably, embryo implantation is a complex and well-orchestrated initial step in the establishment of a healthy pregnancy [210], and a successful implantation requires two conditions to be met: (1) a functional embryo at the blastocyst developmental stage, and (2) a receptive endometrium for proper embryo apposition, adhesion, and invasion [211]. All the abovementioned phenomena are also indicative of a successful pregnancy rate, even if little is known about how a high-fat diet affects them during embryo implantation.

Firstly, a functional blastocyst relies on its membrane lipid profile which, in the context of a high-fat diet, is compromised. Studies in mice suggested that preimplantation embryos are sensitive to high levels of fatty acids, and the early embryo appears to respond to these compromised levels by upregulating the ER stress pathways, leading to downstream effects on preimplantation development [212,213]. Moreover, significant impairment of bovine embryo quality after in vitro culture in fat-enriched conditions led authors to suggest that blastocysts isolated from obese mothers might be more sensitive to oxidative stress [214], resulting in a decreased preimplantation embryo developmental capability and an increased incidence of cell death. Furthermore, this literature survey did not identify a correlation between a high-fat diet and variations in the expression of some genes involved in lipid metabolism in preimplantation embryos, such as Acyl-CoA Synthetase Long Chain Family Member 3 (ACSL3), ELOVL fatty acid elongase 5 (ELOVL5), and ELOVL fatty acid elongase 6 (ELOVL6) [215], suggesting that the mechanisms underlying incorrect implantation in conditions of high dietary intake of fatty acids are still under investigation.

Secondly, endometrial receptivity plays a crucial role in the establishment of a healthy pregnancy, and it relies on a complex process orchestrated by the synergistic actions of the main reproductive hormones, estrogens, and progesterone, as well as other endocrine, paracrine, and autocrine factors [216,217,218].

To ensure that the endometrium is ready to embed the embryo, the decidualization process plays a key role. More in detail, decidualization refers to the functional and morphological changes that occur within the endometrium to form the decidual lining into which the blastocyst implants [219].

Impaired endometrial receptivity is thought to be one of the major reasons for embryo implantation failure [220], and this phenomenon could be due to an impaired decidualization process promoted by a high-fat diet. The mechanism by which a high-fat diet may impair endometrial cellular decidualization is likely multifactorial, although only a few studies are reported in the literature [221]. Nevertheless, a study conducted by Rhee et al. highlighted a correlation between impaired autophagy of endometrial stromal cells (ESCs) and a decreased decidualization process as a result of an imbalance of energy metabolism due to dietary fat intake in a mouse model [180].

This association corroborates the hypothesis that the impairment of these interconnected phenomena could be one of the mechanisms that lead to a poor reproductive outcome and early pregnancy loss in females with high-fat diet habits [222]. To continue, a decreased endometrial decidualization was also observed in obese women due to high-fat habits [223,224,225,226]. The pathogenesis of this phenomenon can consist of a high level of proinflammatory cytokines and an overexpression of pathways related to immune response and ROS production, inducing endothelial dysfunction [223]. It also affects haptoglobulin, an inflammatory marker whose endometrial levels of expression increase in women with high-fat diet habits, resulting in a poor pregnancy rate [225]. Interestingly, the dysregulation of leptin pathways due to dietary fat intake [227,228] seems to be responsible for a decreased expression of matrix metalloproteinase 9 (MMP9), essential in decidualization and embryo invasion by degradation/remodeling of the ECM of the endometrium during implantation [229].

In summary, these data support the thesis that infertility in individuals who eat a high-fat diet is sustained by an unfavorable intrauterine milieu and impaired endometrial receptivity, although more efforts are needed to fully understand the profound mechanisms that govern a reduced endometrial receptivity.

### 5.5. High-Fat Diet Impact on Early Embryogenesis

Timely and finely regulated cleavage cellular events upon zygote formation dictate full completion of embryogenesis leading to live offspring. Any perturbations of these events determine embryo developmental defects, and literature studies concluded that the origin of these developmental problems could arise from compromised oocyte quality [53,230]. As mentioned above, metabolic fluctuations in the follicular environment are associated with deep changes in oocytes and their subsequent developmental competence in the blastocyst stage as shown in different mammal models (non-human primates: [86]; sheep: [118]; mice: [38,41,132,213]; humans: [79]; bovines: [182]; and rabbits: [18]).

The impact of an abnormal maternal metabolic environment on pregnancy outcomes was initially reported some decades ago by means of diabetic mouse models. Female mouse models with type-1 diabetes displayed poor reproductive outcomes, including growth restriction and congenital anomalies [231,232] which were found to be the result of an oocyte maternal effect. Indeed, the authors found that blastocysts derived from diabetic mothers transferred to control non-diabetic females failed to rescue the embryonic developmental defects [232]. Likewise, metabolic fluctuations induced by a high-fat diet were shown to negatively impact early embryo development, affecting pregnancy outcomes. Indeed, high-fat diet mouse models exhibit embryonic developmental defects [155,233] and growth retardation [234]. Similar results were obtained using the rat model [235]. Recently, and in agreement, a prospective study conducted on 240 infertile women showed that high dietary fat consumption was adversely related to embryonic development [236]. Moreover, recent studies are now elucidating that the impaired embryo developmental competence could be due to three main oocyte-related effects: (1) lipotoxicity, (2) mitochondrial dysfunction, and (3) epigenetic dysregulation [53].

Interestingly, an increased oocyte lipid content has been shown in bovine [237] and mouse [157] oocytes exposed to the lipid-rich FF of women with dietary fat habits. This increased content affected the maturation performance and the blastocyst rate formation [157,237]. Moreover, altered oocyte lipid content was also observed in women who normally followed a high-fat diet [238]. More in detail, two clinical studies reported that blastocysts derived from women with high dietary fat intake exhibited increased lipid content and altered metabolism compared with blastocysts from lean women [238,239].

The mechanisms by which high oocyte–lipid exposure is harmful to early embryo developmental capability is thought to be via the induction of oxidative stress and mitochondrial dysfunction, a theory supported by a considerable quantity of work examining the effects of maternally driven regulatory pathways on embryo developmental power [240].

Notably, elevated levels of ROS, indicative of oxidative stress and consequent mitochondrial disfunctions [80], were present in oocytes of mouse models fed with a high-fat diet [134,176,185,187,241,242]. It was also found that manipulating oocytes in vitro by overexpressing the mitochondrial deacetylase Sirtuin 3 (SIRT3) or the glycolysis and apoptosis regulator TP53 Induced Glycolysis Regulatory Phosphatase (TIGAR) in high-fat diet oocytes [242], led to a reduction in ROS and improved oocyte maturation in high-fat-diet obese mice, finally leading to improved embryo preimplantation and development [134,185,187,242]. ROS levels in oocytes of obese mice were also reduced by either in vivo or in vitro treatment with melatonin, via modulation of SIRT3-Superoxide dismutase 2 (SIRT3-SOD2) signaling, which also improved embryo development rates [187]. Interestingly, a recent study supported the idea that supplementing embryo culture media with antioxidant compounds could reduce ROS production, improving the success of embryo development and progression rates in female mice with high-fat diet habits [43].

Lastly, a maternal high-fat diet could also have negative effects on embryo progression through epigenetic dysregulation resulting in an alteration of the DNA methylation status of imprinted genes, metabolism- [40,176,184,243] and developmental- [39,244] related genes. More in detail, oocytes of mice fed high-fat diets displayed reduced global 5-methylcytosine (5 mC), abnormal histone methylation levels [176], and differential methylation of metabolism-related gene loci [40,184]. Furthermore, in a study conducted by Han et al., it a reduction in STELLA (or developmental pluripotency associated 3 [DPPA3]) at gene and protein levels in oocytes from high-fat diet mice [39], a well-recognized maternal factor whose expression begins during primordial germ cell (PGC) specification, resumes exclusively in oocytes, and persists during embryo cleavages was identified [39]. As demonstrated, STELLA deficiency in the oocyte may represent a key connection between maternal metabolic syndrome, embryo development, and, potentially, alterations in offspring physiology [39]. STELLA is critical for normal embryo development by preventing premature TET3 dioxygenase-mediated catalysis of 5 mC to 5-hydroxymethylcytosine (5 hmC) in the maternal zygotic pronucleus [244]. Thus, reduced STELLA protein in oocytes from females with high-fat dietary habits caused abnormal TET3 accumulation in the maternal pronucleus, resulting in premature hydroxylation of 5 mC to 5 hmC [39]. The relevant role of STELLA was demonstrated by microinjecting oocytes of high-fat diet mice with DPPA3 mRNA, which normalized zygote methylation patterns and early embryo development [39].

In summary, all these studies revealed that multiple regulatory pathways are deregulated in embryos derived from oocytes exposed to dysregulated high-fat signals during their development. However, the long-term consequences of each of these perturbations on specific aspects of embryo physiology remain to be fully investigated.

**Figure 4:**
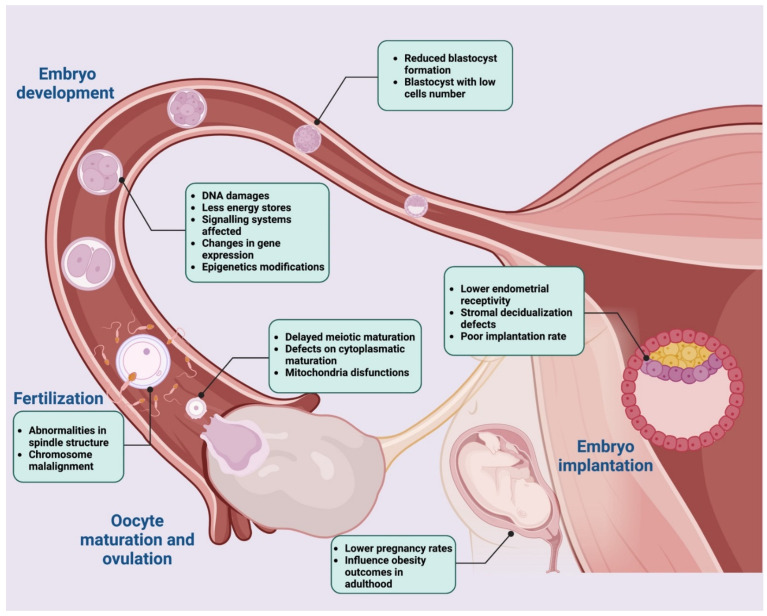
Impact of a high-fat diet on the oocyte developmental competence: from the meiotic maturation to fertilization and early embryo stages. A high-fat diet has a negative impact on oocyte quality, lowering the fertilization potential and interfering with embryo implantation-related events. Three key processes, such as oocyte meiotic maturation, fertilization, and embryo implantation are deregulated in high-fat diet conditions because of poor oocyte quality. Mitochondrial dysfunctions derived from lipotoxic effects of high fat intake causes a delayed/defective oocyte meiotic maturation. Abnormality in the spindle structure as well as in the chromosome alignment impairs fertilization events. Defects in the cross talk between the endometrium and the embryo lower embryo implantation success with a negative impact on pregnancy outcome. Image created with Biorender.com.

The overall flowchart of the data summarized above is reported in Figure 4.

## 6. Discussion and Conclusions

### Overall Sum Up, Key Points and Future Directions

Obesity is a growing health issue for the human population worldwide. Despite the fragmentary source of data collected to date, a comparative analysis of the available findings obtained from several mammal models (Table 1 and Table 2) seems to confirm that obesity negatively impacts the multi-step process controlling the reproductive function. In addition to reproduction recognized redundant mechanisms that converge in guaranteeing the end point of such a key biological function, obesity has been recognized to induce adverse effects on its outcomes affecting estrus/menstrual functions, ovulation, fertilization, pregnancy, and offspring health.

Several studies point to the direct damage caused by a high-fat diet on the early stage of folliculogenesis. Studies also emphasize the depletion of female gamete reserve as a potential mechanism of infertility by supporting, at the same time, the hypothesis of a long-term effect of diet on female reproductive health.

The limited knowledge of the high-fat diet impact on female fertility may be augmented by taking advantage of the reproductive biotechnologies advances that enable us to mimic, with a great level of standardization, several reproductive functional steps (follicle growth, oocyte maturation, fertilization, and early embryo development).

The use of in vitro studies is essential, not only to interpret the results obtained from the in vivo experiments or from clinical data, but also to delineate the mechanisms involved by providing a platform for ovarian cellular/molecular studies within a high-fat enriched diet.

A direct influence of high-fat diet regimes on ovarian follicle functions, for example, was recently elucidated by using in vitro folliculogenesis technology, a valuable experimental model that allows us to reproduce in culture the early stages of follicle development by allowing an analysis of the role exerted on the function of the somatic and/or germinal compartment by mimicking both the intraovarian paracrine and systemic gonadotropin controls in culture. Similarly, assisted reproductive techniques offer high standardized in vitro conditions to demonstrate the adverse impact of high-fat conditions on oocyte maturation and fertilization (IVM/IVF), as well as during embryo development. In addition, modern-engineered reproductive culture systems [246,247,248,249] enable the increasingly physiological in vitro modelling of homeostasis, development, disease, and aging, thus offering novel cultural approaches to better interpret the multifactorial lifestyle impact on fertility. In this context, besides the increasing evidence that inflammation and ovarian aging are implicated in accelerating the decay of fertility, very little data clarify how obesity may further exacerbate these factors in compromising female reproductive health. Unfortunately, the ovary appears to be one of the first organ systems to show the hallmarks of aging, thus suggesting a potential negative interaction of high-fat diet regimes with advanced reproductive age since it may provide a most unfavorable environment for a successful pregnancy. This hypothesis seems to be supported by the progressive decline of ovarian function advancing in both reproductive age and high-fat diet regimes with a common decrease in the quantity and quality of oocytes.

However, data also collected from advanced in vitro models should be validated by in vivo experimental settings and under clinical conditions where ovarian folliculogenesis and oogenesis are under the control of both neuroendocrine and immune systems [58]. To date, many animal models have been developed to explore the effects of obesity and high-fat diet on reproduction. Small rodents, including rats and mice, are the most widely used to study high-fat diet-related disorders due to their great experimental versatility (Table 1). Rodents offer the experimental advantage of a wide range of molecular tools available and, due to the short life span, the effects of high-fat diets have been evaluated for several generations.

However, the translation value of laboratory rodents in identifying the mechanisms and causes of human reproductive pathology deserves reflection. These preclinical models, indeed, differ from humans in relevant systemic and reproductive physiological aspects. Rodents display a very high level in genomic uniformity and controlled lifestyle variables, but they also differ from humans in the mechanism (estrus instead of menstrual and poly-ovulatory instead of mono-ovulatory) and reproductive cycle timing. More in detail, they present a faster follicular growth phase and shorter oocyte maturation window. They also differ in embryo cleavage and embryonic implantation timing [250]. Unfortunately, both retrospective domestic animal studies and those including high translational mammal models (primates: Table 2) clearly indicate the limited data available to date. Most of them addressed the effect of obesity on the end point of the reproductive cycle: oocyte maturation and fertilization.

Overall, the present review highlights the importance of understanding how a high-fat diet adversely affects female fertility and promotes transgenerational disorders by considering the increasing prevalence of obesity among women of reproductive age. Recognizing the burden of such disorders, it is imperative to better elucidate the complex mechanisms involved that affect the female reproductive structures directly and the metabolism and/or inflammatory status indirectly. A better understanding of mechanisms is, finally, strongly required to delineate possible therapeutic strategies, to develop self-awareness of dietary reproductive risk, and concurrently propose solid and rigorous preventive dietary regimes as well as pharmacological interventions.

## Figures and Tables

**Table 1:** Literature survey on reproductive dysfunctions induced by high diet macronutrient in rodent models.

Rodent Models of Obesity (%)		Diet Macro-Nutrients (~ %)	Morphological Effects on Female Fertility [References]			
			Endocrine	Ovarian Folliculogenesis		Oocyte Competencies
				Granulosa Cells	Theca Cells	
**60%** of mammal models	Mouse (51%)	~44% Fat ~52% Carbohydrate ~20% Protein	**2.** Estrous cycle impairments [33,34,36,75,95,99,100,115,116,117,132,179,207] **3.** Ovary reserve depletion [36,47,81,82,100,129] **4.** Altered follicle gene profiles [33,35,36,50,75,77,100,115] **5.** Gonadotropin release defects [14,33,34,36,37,75,95,99,100,115,132,179,207] **6.** Steroid release impairment [33,36,50,99,100] **7.** Perinatal growth delay [50,89,100,196,241] **8.** Pubertal onset defects [21,50]	**1.** Impaired cell control [34,41,47,54,56,73,74,95] **2.** Estradiol synthesis [34,36,38,41,44,46,47,48,50,56,70,74,75,77,80,83,94,95,96,99,100,179] **3.** Development failures [75,76,77,151,155,156,233] **4.** Lipid droplets [80,154]	**1.** Androgen production dysregulation [34,36,41,44,46,47,48,50,70,74,75,80,94,95,96,99,100,122,179] **2.** LH receptors [44,97,221]	**1.** Oocyte apoptosis [48,99,151,153,154,155,176,179,180,181] **2.** Partial meiotic competence [33,40,46,48,99,100,114,132,176,177,178] **3**. MII oocyte [33,35,37,40,43,46,48,55,76,96,99,100,114,115,117,132,149,153,155,157,176,177,178,180,183,185,207,213,233,234] **4.** Fertilization rate [33,37,40,43,46,114,121,153,155,183] **5.** Oocyte lipid droplets [43,47,55,115,132,145,154,212] **6.** Mitochondrial de-regulation [33,43,47,55,80,100,115,133,145,149,151,176,177,178,179,181,183,184,185,212,241,242] **7.** Spindle defects [46,48,55,100,117,132,176,177,178,179,183,185,187,196,242] **8.** Chromosome’s misalignment [46,100,117,177,178,185,187,196,242] **9.** Epigenetic modifications [39,40,44,47,96,117,176,179,184]
	Rats (9%)	~40% Fat ~66% Carbohydrate ~20% Protein	**1.** Pubertal onset defects [19,25] **2.** Estrous cycle impairments [19,25,26,32,42,71,72,98] **3.** Ovary reserve depletion [19,42,71] **4.** Altered follicle gene profiles [19,25,26,32,42,72,84,98] **5.** Gonadotropin release defects [9,19,25,42,84,98] **6.** Steroid release impairment [19,25,26,84,98] **7.** Perinatal growth delay [27]	**1.** Impaired cell control [26,32,42,71,72,235] **2.** Estradiol synthesis [2,19,24,25,26,32,42,71,84,98]	**1.** Androgen synthesis dysregulation [19,25,26,32,42,71,72,84,98] **2.** LH receptors [25]	**1.** Oocyte apoptosis [42,195] **6.** Mitochondrial de-regulation [42]

**Table 2:** Literature survey on reproductive dysfunctions induced by high diet macronutrient in mammal non-rodent models.

Non-Rodent Models of Obesity (% of Mammal Models)		Diet Macro-Nutrients (~ %)	Morphological Effects on Female Fertility [References]			
			Endocrine	Ovarian Folliculogenesis		Oocyte Competencies
**POLI** **GASTRIC** (15%)	Ovi- caprine (3%)	n.s^*^ Fat n.s^*^ Carbohydrate ~17% Protein	**2.** Estrous cycle impairments [78,118] **9.** Ovulation timing changes [78,118,148]	**3.** Development failures [78]	**3.** Theca dysfunction [78]	**4.** Fertilization rate [118,148]
	Bovine (12%)	60% Fat; ~57% Carbohydrate ~88% Protein	**1.** Pubertal onset defects [20] **2.** Estrous cycle impairments [112,182,186] **3.** Ovarian reserve depletion [182,186]	**3.** Development failures [20,150,159,160,161,162,163,164,165,166,167,168]	**3.** Theca dysfunction [20] **4.** Late folliculogenesis defects mediated by angiogenesis [112]	**1.** Oocyte apoptosis [182] **3.** MII oocytes [20,150,159,160,161,162,163,164,165,166,167,168,175,182] **4.** Fertilization rate [150,182,186] **5.** Oocyte lipid droplets [237]
**MONO** **GASTRIC** (**5%**)	Pig (4%)	~62% Fat; ~20% Carbohydrate	**5.** Gonadotropin release defects [28,29] **6.** Steroid release defects [28,29]	**2.** Estradiol synthesis [28,29,45,85] **3.** Development failures [85,169,170,171]	**3.** Theca dysfunction [28,29,45]	**2.** Partial meiotic competence [45,85] **3.** MII oocytes [45,85]
	Rabbit (1%)	~8% Fat n.s^*^ Carbohydrate n.s^*^ Protein	**3.** Ovarian reserve depletion [18]	**3.** Development failures [18]	**3.** Theca dysfunction [18]	-
**PRIMATES** (**20%)**	Human (17%)	n.s^*^	**3.** Ovarian reserve depletion [128] **5.** Gonadotropin release defects [79] **6.** Steroid release defects [79]	**3.** Development failures [5,79,92,93,158] **4.** Lipid droplets [79] **5.** Altered angiogenesis [102,103,104,105,106,107,108,109,110] **6.** FF oxidative biomarkers [147,148,152,153,203]	**3.** Theca dysfunction [79,119]	**3.** MII oocytes [4,79,152,236,237,238,239] **4.** Fertilization rate [4,79,225,237,238]
	Non-human (3%)	~35% Fat ~49% Carbohydrate ~18% Protein	**2.** Estrous cycle impairment [30,57,86,87,245] **4.** Altered follicle gene profiles [30,57,86,87] **5.** Gonadotropin release defects [30,57,86,87] **6.** Steroid release defects [30,57,86,87] **9.** Ovulation timing changes [30,57,86,87]	**2.** Estradiol synthesis [30,57,86,87]	**3.** Theca dysfunction [57,86,87]	**3.** MII oocytes [57,86,87] **4.** Fertilization rate [57,86,87]

n.s^*^ concentration not specified.

## Data Availability

Not applicable.
